# Supplementary material for: Resolving the full spectrum of human genome variation using Linked-Reads
Source: Genome Res. 2019 Apr;29(4):635–45. doi: 10.1101/gr.234443.118 (PMC6442396; doi:10.1101/gr.234443.118)
Supplement: Supplemental Material [file supp_gr.234443.118_Supplemental_longranger-2.2.zip › longranger-2.2.tar.bz2!/lr2.2-public/lib/stan/cmdstan/stan/doc/api/html/agrad_2rev_2matrix_2trace__gen__inv__quad__form__ldlt_8hpp_source.html]

Stan: src/stan/agrad/rev/matrix/trace\_gen\_inv\_quad\_form\_ldlt.hpp Source File


|  |  |
| --- | --- |
|  | Stan  2.5.0  probability, sampling & optimization |


- Main Page
- Related Pages
- Namespaces
- Classes
- Files

- File List
- File Members

All Classes Namespaces Files Functions Variables Typedefs Enumerations Enumerator Friends Macros Pages

- src
- stan
- agrad
- rev
- matrix

trace\_gen\_inv\_quad\_form\_ldlt.hpp

Go to the documentation of this file.

1 #ifndef STAN\_\_AGRAD\_\_REV\_\_MATRIX\_\_TRACE\_GEN\_INV\_QUAD\_FORM\_LDLT\_HPP

2 #define STAN\_\_AGRAD\_\_REV\_\_MATRIX\_\_TRACE\_GEN\_INV\_QUAD\_FORM\_LDLT\_HPP

3

4 #include <stan/math/matrix/Eigen.hpp>

5 #include <stan/agrad/rev/var.hpp>

6 #include <boost/type\_traits/is\_same.hpp>

7 #include <boost/utility/enable\_if.hpp>

8 #include <stan/math/error\_handling/matrix/check\_multiplicable.hpp>

9 #include <stan/math/error\_handling/matrix/check\_square.hpp>

10 #include <stan/agrad/rev/matrix/trace\_inv\_quad\_form\_ldlt.hpp>

11

12 namespace stan {

13  namespace agrad {

14

20  template <typename T1,int R1,int C1,typename T2,int R2,int C2,typename T3,int R3,int C3>

21  inline typename

22  boost::enable\_if\_c<boost::is\_same<T1,var>::value ||

23  boost::is\_same<T2,var>::value ||

24  boost::is\_same<T3,var>::value, var>::type

25  trace\_gen\_inv\_quad\_form\_ldlt(const Eigen::Matrix<T1,R1,C1> &D,

26  const stan::math::LDLT\_factor<T2,R2,C2> &A,

27  const Eigen::Matrix<T3,R3,C3> &B)

28  {

29  stan::math::check\_square("trace\_gen\_inv\_quad\_form\_ldlt(%1%)",D,"D",

30  (double\*)0);

31  stan::math::check\_multiplicable("trace\_gen\_inv\_quad\_form\_ldlt(%1%)",A,"A",

32  B,"B",(double\*)0);

33  stan::math::check\_multiplicable("trace\_gen\_inv\_quad\_form\_ldlt(%1%)",B,"B",

34  D,"D",(double\*)0);

35

36  trace\_inv\_quad\_form\_ldlt\_impl<T2,R2,C2,T3,R3,C3> \*\_impl = new trace\_inv\_quad\_form\_ldlt\_impl<T2,R2,C2,T3,R3,C3>(D,A,B);

37

38  return var(new trace\_inv\_quad\_form\_ldlt\_vari<T2,R2,C2,T3,R3,C3>(\_impl));

39  }

40

41

42  }

43 }

44

45 #endif

\_impl

quad\_form\_vari\_alloc< TA, RA, CA, TB, RB, CB > \* \_impl

**Definition:** quad\_form.hpp:118

check\_multiplicable.hpp

stan::math::check\_multiplicable

bool check\_multiplicable(const char \*function, const T1 &y1, const char \*name1, const T2 &y2, const char \*name2, T\_result \*result)

**Definition:** check\_multiplicable.hpp:15

stan::math::LDLT\_factor

**Definition:** LDLT\_factor.hpp:16

trace\_inv\_quad\_form\_ldlt.hpp

var.hpp

Eigen.hpp

stan::agrad::var

Independent (input) and dependent (output) variables for gradients.

**Definition:** var.hpp:27

stan::agrad::trace\_gen\_inv\_quad\_form\_ldlt

boost::enable\_if\_c< boost::is\_same< T1, var >::value||boost::is\_same< T2, var >::value||boost::is\_same< T3, var >::value, var >::type trace\_gen\_inv\_quad\_form\_ldlt(const Eigen::Matrix< T1, R1, C1 > &D, const stan::math::LDLT\_factor< T2, R2, C2 > &A, const Eigen::Matrix< T3, R3, C3 > &B)

Compute the trace of an inverse quadratic form.

**Definition:** trace\_gen\_inv\_quad\_form\_ldlt.hpp:25

stan::math::check\_square

bool check\_square(const char \*function, const Eigen::Matrix< T\_y, Eigen::Dynamic, Eigen::Dynamic > &y, const char \*name, T\_result \*result)

Return true if the specified matrix is square.

**Definition:** check\_square.hpp:24

check\_square.hpp


---

[ Stan Home Page ]

*© 2011–2014,
Stan Development Team.*
